# Association of Sensory Processing and Eating Problems in Children with Autism Spectrum Disorders

**DOI:** 10.1155/2011/541926

**Published:** 2011-09-22

**Authors:** Geneviève Nadon, Debbie Ehrmann Feldman, Winnie Dunn, Erika Gisel

**Affiliations:** ^1^École de Réadaptation, Faculty of Medicine, University of Montreal, Montreal, QC, Canada H3N 1H1; ^2^École de Réadaptation, Centre for Interdisciplinary Research in Rehabilitation (CRIR), Montreal, QC, Canada H2H 2N8; ^3^Department of Occupational Therapy Education, University of Kansas Medical Center, Kansas City, KS 66160, USA; ^4^Faculty of Medicine, School of Physical and Occupational Therapy, McGill University, Montreal, QC, Canada H3G 1Y5

## Abstract

“Selective” or “picky eating” is a frequent problem in children with autism spectrum disorders (ASD). Many of these children do not treat sensory input, particularly olfactory, auditory, visual, and tactile information in the same manner as their typically developing peers of the same age. The purpose of this paper was to examine the relationship between problems of sensory processing and the number of eating problems in children with ASD. Of 95 children with ASD, 3 to 10 years of age, 65 percent showed a definite difference and 21 percent a probable difference in sensory processing on the total score of the *Short Sensory Profile*. These results were significantly related to an increase in the number of eating problems measured by the *Eating Profile*. These results could not be explained by age, sex, mental retardation, attention deficit disorder, or hyperactivity. Timely interventions focusing on the sensory components of eating must now be developed.

## 1. Introduction

Approximately 25% of all children experience eating problems during the early years of life, but this number may rise to as high as 80% in children with developmental difficulties [1, 2]. “Selective” or “picky eating,” defined as eating a limited variety of food and refusal to eat or taste new foods [2, 3], is a frequent problem in children with autism spectrum disorders (ASD; [4–10]). These behaviours of selective or picky eating may vary greatly, but are considered a problem when they interfere with the child's daily routine or social integration.

The frequency of “selective eating” by either food type or texture is significantly higher in children with ASD than in typically developing children [6, 7, 9–14]. One of the reasons may be that these children have specific developmental delays which may also affect eating. Difficulties with socialization may have an impact on the pleasure of eating in the company of others. This problem may also make learning by imitation and accepting nutritionally balanced meals more difficult. Similarly, having limited interests may restrict intake to known and familiar foods.

Much research has focused on the sensory peculiarities of individuals with ASD, starting with Kanner in 1943 [15]. Individuals with autism have sought to share and communicate their sensory experiences with the public for many years, particularly since the advent of the internet [16–18]. Although sensory processing problems are not universal or exclusive to the population with ASD, many authors agree that they exist in this population [19–24]. In fact, quite a large percentage of children with ASD (78 to 90%) have sensory processing problems [25, 26]. These are mostly problems of sensory modulation expressed as hyper- and/or hyposensitivity [22, 24, 25, 27–29]. Such problems have an impact on the child's development and the ability to perform activities of daily living, such as eating.

A meal is a complex sensory experience consisting of the foods with their appearance, odors, textures, and tastes, as well as the presence of others including the auditory component of their conversations. In addition, motor planning is necessary for postural control and for the manipulation of eating utensils. Oral hypersensitivity is more frequent in children with ASD than in typically developing children [22, 24, 25]. An association between feeding problems and sensory defensiveness has been shown in otherwise typically developing children [30] and has been suggested by Cermak et al. [31] in children with ASD. The problems described by Smith et al. [30] are very similar to what is found clinically in children with ASD; they are “picky” eaters, eat few vegetables, rarely eat the same meal as the rest of the family, do not want different foods to touch each other, have aversions to certain tastes and textures, refuse some foods because of their smell, and do not like extremes of temperature. Literature on intervention for eating problems recognizes the contribution of sensory processing to eating and suggests that it must be taken into consideration [7, 32, 33]. Accordingly, a category named “Sensory Food Aversion” has been included in the *Diagnostic Classification of Mental Health and Developmental Disorders of Infancy and Early Childhood: Revised Edition *(DC:0–3R) [34], and there is currently a proposal to include a diagnosis of “Avoidant/Restrictive Food Intake Disorder,” in the *DSM-V *for individuals who accept only a limited diet with respect to sensory features [35]. The nature and extent to which sensory processing is related to eating problems in children with ASD remain to be determined.

Therefore, the purpose of this study was to establish whether there is a relationship between sensory processing problems and the number of eating problems in children with ASD. We expected that children with sensory processing problems would have more eating problems than those without such problems.

## 2. Methods

### 2.1. Participants

Children with a diagnosis of autism, pervasive developmental disorder not otherwise specified (PDD-NOS), or Asperger syndrome, aged 3–10 years, were eligible for this study. They were registered in one of four local rehabilitation centers, one tertiary paediatric hospital, or one of two parent associations in Quebec, Canada. Exclusion criteria were childhood disintegrative disorder and Rett syndrome.

Families of children identified as eligible by the above criteria were contacted by the personnel of the rehabilitation centers and hospital. We approached parent associations via the web by posting information regarding the study, including a telephone number to call. Parents who indicated an interest in the study were sent a letter explaining the purpose and specifics of the study, as well as a consent form. 

Diagnosis and cognitive status of the children were established either by a child psychiatrist or by a multidisciplinary team (tertiary paediatric hospital). Because this study is multicentered, information was not consistently available concerning assessments used for the diagnosis. Where limited information was available, medical chart review was used to complement the information. 

### 2.2. Measures

A questionnaire was used that had been developed by clinicians and focuses on developmental eating milestones, mealtime behaviours of the child, such as eating autonomy and impact on the daily life of the family [36]. Questions were added to this instrument based on a review of the literature [37], and clinical experience. Face validity was established by five occupational therapists having extensive experience with children with ASD and an adult with high-functioning autism who works as a consultant in this field. Professionals gave their feedback individually to the authors. Minor clarifications were suggested and some more questions were added. The questionnaire was pretested with five parents from diverse educational backgrounds. They provided feedback on the clarity of the questions and the length of the questionnaire. Suggested changes were integrated. 

Eleven domains are covered (145 items). (1) Dietary history of the child (16 items) dealing with feeding and oral behaviours during infancy, changes in the amount of intake during infancy, and the parent's perception of the adequacy of intake; (2) child health (8 items), these questions focus on general health and weight gain over the last 3, 6, and 12 months; (3) family dietary history (7 items) establishes whether there are any food intolerances/allergies or picky eating in other members of the family; (4) mealtime behaviours of the child (23 items) focus on oral-motor skills such as chewing, swallowing, drooling, gagging, coughing, choking, and social skills of eating with the family and staying at the table for the length of the meal; (5) food preferences (19 items) deal with the presentation, characteristics, and appearance of foods; (6) eating autonomy (11 items) focuses on support (utensils, seating) and assistance needed to eat independently; (7) behaviours outside of mealtimes (12 items) look at the compliance and integration in the social milieu; (8) impact on daily life (8 items) looks at the ease/effort with which meals are taken; (9) strategies used to resolve difficulties encountered at mealtimes (31 items), examine behavioural and nutritional approaches taken; (10) communication abilities of the child (8 items) looks at whether needs and intentions can be communicated; (11) socioeconomic factors of the family (2 items). 

Answers to some questions are dichotomized (yes/no), others use a Likert scale of three, four, or five levels (e.g., always/often/rarely/never/nonapplicable). An identification page (12 items) documents demographic information such as date of birth, diagnosis, comorbidities, medication(s), and ethnicity. 

The questionnaire further underwent preliminary testing for test-retest reliability. Good to high agreement was found for most items although there were 4 domains that had kappa values below 0.4, accounted for by low frequency of responses. Further development is anticipated to achieve acceptable psychometric properties for measurement tools. Following these procedures the questionnaire was named the *Eating Profile* [38] and was used for this study. 

The reason for developing this questionnaire was that other existing tools did not sufficiently serve our purpose; that is, address the specific needs of children with ASD, provide a dietary history of the child and parents, focus on the child's autonomy at mealtime, or strategies attempted by parents to address mealtime problems. These issues needed to be addressed to set the stage for future rehabilitative interventions. Further details can be found in a companion publication [11].

**The Short Sensory Profile (SSP)**

The SSP is a standardized questionnaire [39]. It permits clinicians and researchers to quickly identify children with sensory processing problems [40]. The 38 items of the SSP are extracted from the long version of the *Sensory Profile* (SP; 125 items), based on factor analyses and correlation studies from two samples of 117 and 1037 children with a variety of problems [40]. The SSP consists of 7 sections: (1) tactile sensitivity (7 items), (2) taste/smell sensitivity (4 items), (3) movement sensitivity (3 items), (4) underresponsive/seeks sensation (7 items), (5) auditory filtering (6 items), (6) low energy/weak (6 items), and (7) visual/auditory sensitivity (5 items). The internal reliability, using Cronbach's alpha, for the test total and sections ranges from .70 to .90 [39].

The SSP is filled out by the caregiver and takes approximately 10 minutes to complete. A sample question is “reacts emotionally or in an aggressive manner to touch.” The caregiver determines how often the statement occurs (always, often, sometimes, rarely, never). Answers are summed in each section and a total score is calculated. “Typical” responses are within 1SD of the mean, “probable differences” lie between the first and second SD below the mean and a “definite difference” will lie below the 2SD mark. Construct validity has been established [40]. Internal consistency ranges from 0.25 to 0.75 [40]. The SSP was translated into French by the first author and back translated into English by an English speaking occupational therapist. The 2 English versions were compared by three authors (G. Nadon, D. E. Feldman, and E. Gisel) to examine the concordance of the 2 versions. A few French words were changed for a closer fit with the original intent of the statements. The French version was then approved by the publisher and a distribution permit was obtained (Harcourt Assessment Inc.). 

### 2.3. Procedures

The *Eating Profile* [38] and the SSP [39] were sent to parents. A few days following receipt of the questionnaires a research assistant (occupational therapist) called parents to clarify any questions they might have regarding the completion of the questionnaires. Only factual information was provided in order not to influence parents' responses. Filling out the 2 questionnaires took about one hour. All questionnaires were returned by mail to the authors.

Of 119 families recruited, 12 did not return the questionnaires and 1 response was incomplete. Eleven children, older than 10 years, were excluded from analyses, because the SSP is standardized for children 3 to 10 years of age, leaving a total sample of *n* = 95. All parents gave consent and children older than seven years gave assent for parents to answer the questionnaires of the study. Parents had to understand and read French or English. The study was approved by the research ethics board of the Montreal Children's Hospital and the research committees of the four local rehabilitation centers.

### 2.4. Analyses

Data reduction used 60 (out of 145) responses from six domains of the *Eating Profile. *These were identified as indicative of feeding problems and were retained for further analysis (domain 1: dietary history of the child; domain 2: child health; domain 4: mealtime behaviours of the child; domain 5: food preferences; domain 6: eating autonomy, and domain 8: impact on daily life). These responses were chosen based on clinical experience and the literature review [37]. The following are some examples of questions retained: *Was the transition from thin puree to textured food easy for your child? At the present time, does your child's diet include more than 20 different foods? (including liquids). Depending on the person present, does your child's eating vary? (e.g., eats apples only with daddy). Do you have to prepare a different meal for your child? *The remaining domains were not used as they do not directly address the child's current eating problems. Means and standard deviations were calculated for continuous variables, and proportions for categorical variables from the 60 responses.

Analysis of variance was used to compare differences in the three categories of the SSP (typical, probable, and definite problem). Linear regression analyses were performed to determine any association between eating problems and sensory processing, controlling for age, diagnostic category, mental retardation (MR), attention deficit disorder (ADD), and hyperactivity (HYPER). The contribution of these last 3 conditions was controlled for in the multiple linear regression analysis to determine their possible contribution to our outcome measures. None of the other associated conditions were entered into the linear regression model (see Table 1), because there were not enough children with these conditions. The dependent variable was the number of eating problems and the independent variable was the classification (typical, probable, and definite problem) on the SSP. Diagnostic category was dichotomized as autism versus PDD-NOS and Asperger syndrome.

## 3. Results

The mean age of the group was 7.3 ± 2.5 years, and 87 (91.6%) of the 95 children were males. Sixty-one percent had a diagnosis of autism, 29% pervasive developmental disorder-not otherwise specified (PDD-NOS), and 10% had Asperger syndrome. Table 1 describes the sample in terms of demographics and comorbidities. 

Children with ASD had at least one other associated medical condition; the most common conditions were attention deficit disorder (23%), hyperactivity (22%), and mental retardation (23%). Medications were carefully reviewed with respect to their effects on food intake. Twenty-three children took medications that may suppress food intake (Ritalin, Concerta, Adderall, Keppra, and Strattera). Seven parents of these 23 children expressed concern regarding the quantity of their children's food intake. Five parents indicated that they regarded their child's weight as below average. In 6 cases health was rated average to poor. 

Table 2 illustrates the 7 sections of sensory responses on the SSP and their association with the mean number of eating problems in children with ASD. 

Overall, children with “definite” sensory problems had significantly more eating problems than those with “typical” performance as born out in the significant difference between these groups in the total score. Of the 7 sections examined, children with tactile sensitivity (linear regression, *F*_2,91_ = 4.04; *P* < 0.021), taste and smell sensitivities (linear regression, *F*_2,91_ = 28.83, *P* < 0.0001), as well as visual/auditory sensitivities (linear regression, *F*_2,92_ = 5.49, *P* < 0.006) had significantly more eating problems than children with typical performance. Roughly half to two-thirds of children in the 3 significant sections had either definite or probable sensory processing problems in association with their eating problems. These children had a mean of 17 eating problems compared to a mean of 11 problems in children with typical performance. 

We calculated multiple linear regression models for each of the seven sections of the SSP as well as the total score (Table 3). The dependent variable was the number of problematic eating behaviors, and the independent variable was having a definite problem on the SSP. Covariates were age, diagnostic category (autism versus PDD-NOS or Asperger syndrome), and the three conditions of MR, ADD, and HYPER because they could contribute to feeding problems in and of themselves. For the total score of the SSP and for three sections (taste/smell sensitivity; auditory filtering; visual/auditory sensitivity), having a definite problem was significantly associated with a greater number of eating problems. Although not statistically significant (*P* = 0.066), there was a tendency for tactile sensitivity to be associated with the number of eating problems.

## 4. Discussion

Many children with autism spectrum disorders do not treat sensory input in the same manner as their typically developing peers of the same age, particularly tactile, olfactory, and visual/auditory information, as measured by the SSP [39]. On our total score, sixty-five percent of the children with ASD showed a definite difference and twenty-one percent a probable difference.

Our results suggest that certain sensory modalities may influence the number of feeding problems more than others. For example, children who were classified in the “definite difference” category on “tactile sensitivity” showed problems with drooling, the social behaviours at mealtime, as well as having unusual food preferences with respect to commercial brands, specific recipes, color, texture, or temperature of the food [11]. These findings support an association of tactile defensiveness and food selectivity reported by Smith et al. [30] and suggested by Cermak et al. [31] in children with ASD. Exploration through touching is a preliminary step to the introduction of new foods in young children [32, 33]. Children showing sensory defensiveness might be less inclined to explore foods with their hands. Others may have difficulties with the feel of utensils, the close presence of other children, or the routine clean-up after a meal. 

Results similarly indicated that children with ASD and problems with taste and/or smell sensitivity have mealtime problems. Similar to the section on tactile sensitivity, there were problematic mealtime behaviours but even more pronounced food preferences. This affected the eating autonomy of children more than tactile sensitivity, particularly in eating without assistance and using eating utensils, such as a fork. Auditory filtering affected these behaviours to the same extent as taste/smell sensitivities. This confirms that eating is indeed a complex multisensory experience. How these issues may be addressed in therapy remains to be determined. 

Although the section “under responsive/seeks sensation” was not significantly associated with the number of eating problems, this may be due to the lowest mean number of eating problems in the group with a “definite difference,” compared to the sections that showed a significant difference. Nonetheless, our analysis of problematic behaviours as measured by the *Eating Profile* shows that the magnitude of the problems is similar to the sections discussed above. At present we are not able to differentiate “underresponsive” from “overresponsive” children who manifest sensory sensitivities. The characterization of these groups, with respect to “responsiveness,” will be essential before any therapeutic approaches can be considered. 

A significant association was also found between visual and auditory sensitivity and the number of eating problems in children with ASD. Mealtimes can indeed be noisy during the preparation of food, the manipulation of utensils, and ongoing conversations. Even the sound of their own chewing can bother some very sensitive children. Whether at school, or in child care, the noise level is usually above the one experienced in a child's home. However, the problematic mealtime behaviours in this section were associated with going through special phases in a child's eating habits, drooling, lacking appetite, and having fewer than 20 different foods in their dietary repertoire. As before, there were strong aversions to specific characteristics of foods. Children with visual sensitivities may react more to the visual stimuli of foods which may evoke unpleasant memories of their taste or texture. In typically developing children the visual exploration of food may facilitate the expectation of their taste/texture and thereby ease the acceptance of new foods. 

The sections of “movement sensitivity” and “low energy/weak” showed problematic mealtime behaviours that were similar to all other sections. The strongest component was again food preferences, with little impact on eating autonomy, but a stronger impact on daily life. 

 The strength of this study is the size of its sample, where observations from 95 families contributed to our understanding of the daily challenges experienced by parents and children with ASD. It is the first study examining the association of eating behaviors with sensory processing in children with ASD. The study also determined that some of the major associated conditions with ASD (MR, ADD, HYPER) in and of themselves cannot explain this unique sensory behavior and the number of eating problems observed in this population. 

One of the limitations of any study of mealtime behaviours is the fact that a psychometrically sound tool has yet to be fully developed [41, 42]. The *Eating Profile* [38] underwent preliminary testing and showed adequate face and content validity, as well as satisfactory test-retest reliability. Further development is anticipated to achieve acceptable psychometric properties for clinical measurement tools. 

A direct observation of mealtimes would have been desirable to validate observations from parent reports. However, valid information is difficult to obtain because the presence of an observer (or camera) may influence or change the routine behaviours of the observed “subjects.” Keeping of a daily log of a meal may aid the validation of both intake and a child's behaviours at the meal. In this regard, Herndon et al. [43] found few differences in average nutrient intake between children with ASD compared to typically developing peers based on a 3-day diet record. 

This study used a convenience sample and it will not be possible to determine if it is representative of all children with ASD and eating problems in this geographic region. It is possible that participating parents were particularly concerned about their child's eating behaviors and were looking for guidance and direction from the investigators. The main objective of this study was not to establish the prevalence of specific eating problems in this population but to gain a better understanding of the relation between sensory processing and eating problems when present. 

## 5. Conclusion

This study examined the association of eating behaviors with sensory processing. Close to 90% of preschool and school-age children with ASD do not treat sensory input, particularly tactile, olfactory, visual, and auditory information in the same manner as their typically developing peers of the same age. An association of sensory processing problems and number of eating problems was documented. The frequency and severity of eating problems perceived by parents in their children with ASD highlights the need for systematic evaluation of this activity of daily living in conjunction with the sensory processing issues associated with food preferences and their impact on daily life. A definite diagnosis of ASD is often only established between the ages of 2 and 4 years, and so parents are frequently left struggling on their own trying to deal with many problems, including eating. We propose that an examination of mealtime behaviours be part of the diagnostic workup, including a sensory profile, in order to provide guidance to caregivers and parents.

## Figures and Tables

**Table 1:** Characteristics of the sample (children with ASD; *n* = 95).

Variable	ASD *n* (%)
Male	87 (91.6)
Age	
3–5 yrs	47 (49.5)
6–10 yrs	48 (50.5)
Nationality	
French Canadian^a^	83 (87.4)
English Canadian	9 (9.5)
Other Nationality	15 (15.8)
Diagnosis	
Autism	58 (61.1)
PDD-NOS	28 (29.4)
Asperger syndrome	9 (9.5)
Associated conditions	
Attention deficit disorder	22 (23.2)
Mental retardation	22 (23.2)
Hyperactivity	21 (22.1)
Learning disability	10 (10.5)
Allergies	15 (15.8)
Lactose intolerance	10 (10.5)
Pulmonary problems	8 (8.4)
Diarrhea	6 (6.3)
Epilepsy	2 (2.1)
Constipation	3 (3.2)
Failure to thrive	4 (4.2)
Anxious behaviors	2 (1.9)
Coprophagia	2 (1.9)
Visual Problems (wears glasses)	2 (1.9)
Cardiac problems	1 (1.1)
Metabolic disorders	1 (1.1)
Gastroesophageal reflux	1 (1.1)
Other(s)	0

^a^The sum of nationalities is greater than 100% because some children have more than one nationality.

**Table 2:** Classification of children with ASD, by SSP and mean number of associated eating problems.

Sections of the SSP and children's classifications	ASD *n* (%)	Mean number of feeding problems (SD)	*P* ^ b^
Tactile sensitivity^a^			
Definite difference	35 (36.8)	15.6 (6.4)	
Probable difference	23 (24.2)	15.4 (4.7)	<0.021
Typical performance	36 (37.9)	11.8 (6.7)	
Taste/smell sensitivity^a^			
Definite difference	46 (48.4)	18.0 (5.2)	
Probable difference	15 (15.8)	11.5 (4.7)	<0.0001
Typical performance	33 (34.7)	9.7 (4.9)	
Movement sensitivity			
Definite difference	27 (28.4)	15.0 (6.7)	
Probable difference	19 (20.0)	14 (5.7)	0.637
Typical performance	49 (51.6)	13.6 (6.4)	
Underresponsive/seeks sensation			
Definite difference	64 (67.4)	14.7 (5.9)	
Probable difference	16 (16.8)	12.2 (7.1)	0.336
Typical performance	15 (15.8)	13.5 (7.1)	
Auditory filtering			
Definite difference	53 (55.8)	15.3 (6.6)	
Probable difference	23 (24.2)	12.7 (4.8)	0.123
Typical performance	19 (20.0)	12.5 (6.8)	
Low energy/weak			
Definite difference	41 (43.2)	15.2 (6.9)	
Probable difference	12 (12.6)	11.8 (5.2)	0.223
Typical performance	42 (44.2)	13.7 (5.9)	
Visual/auditory sensitivity			
Definite difference	21 (22.1)	17.6 (6.1)	
Probable difference	30 (31.6)	14.2 (6.5)	<0.006
Typical performance	44 (46.3)	12.3 (5.7)	
Total score			
Definite difference	62 (65.3)	15.5 (6.0)	
Probable difference	20 (21.1)	10.9 (6.0)	<0.007
Typical performance	13 (13.7)	12.2 (6.3)	

^a^ *n* = 94, 1 data point missing.

^b^Comparison of “definite and probable” group with typical performance.

**Table 3:** Results of multiple linear regression analysis: Association between number of problematic eating behaviors with sensory problems (adjusted for age, diagnosis, mental retardation, Attention Deficit Disorder & Hyperactivity: Autism versus PDD-NOS & Asperger Syndrome).

	ß (se)	*P*-value
Tactile sensitivity (definite)	2.50 (1.34)	0.066
Age (unit increase)	−0.53 (0.32)	0.099
Diagnosis (autism)	1.11 (1.34)	0.406
Mental retardation	1.56 (1.59)	0.330
Attention deficit disorder	−3.10 (2.55)	0.229
Hyperactivity	2.35 (2.60)	0.367
Taste/smell sensitivity (definite)	7.42 (1.08)	<0.001
Age (unit increase)	−0.27 (0.26)	0.318
Diagnosis (autism)	0.76 (1.08)	0.487
Mental retardation	0.78 (1.33)	0.561
Attention deficit disorder	−1.25 (2.11)	0.557
Hyperactivity	0.10 (2.17)	0.963
Movement sensitivity (definite)	1.96 (1.46)	0.182
Age (unit increase)	−0.59 (0.33)	0.074
Diagnosis (autism)	0.94 (1.32)	0.482
Mental retardation	1.75 (1.59)	0.276
Attention deficit disorder	−3.25 (2.57)	0.208
Hyperactivity	2.70 (2.62)	0.306
Underresponsive/seeks sensation (definite)	1.83 (1.40)	0.194
Age (unit increase)	−0.42 (0.33)	0.203
Diagnosis (autism)	0.90 (1.32)	0.499
Mental retardation	2.01 (1.58)	0.206
Attention deficit disorder	−3.33 (2.57)	0.199
Hyperactivity	2.22 (2.61)	0.397
Auditory filtering (definite)	3.12 (1.30)	0.019
Age (unit increase)	−0.50 (0.31)	0.114
Diagnosis (autism)	1.18 (1.30)	0.367
Mental retardation	1.34 (1.57)	0.397
Attention deficit disorder	−4.19 (2.56)	0.104
Hyperactivity	2.99 (2.57)	0.247
Low energy/weak (definite)	2.01 (1.29)	0.123
Age (unit increase)	−0.47 (0.32)	0.145
Diagnosis (autism)	0.88 (1.32)	0.506
Mental retardation	1.99 (1.57)	0.209
Attention deficit disorder	−3.41 (2.57)	0.188
Hyperactivity	2.91 (2.62)	0.270
